# Abstracts and Gleanings

**Published:** 1884-03-20

**Authors:** 


### ABSTRACTS AND GLEANINGS.

**Homeopathy in the British Medical Association.**—Quite a flurry was created in the British Medical Association at the annual meeting in Liverpool several months ago, by a strenuous effort on the part of homeopaths and their friends to gain admittance and recognition. The opposition was so impatient and so violent, and the advocates of admission so persistent and irritable, as to create an uproar not creditable to a scientific body. The question might have been settled without disorder by the weight of numbers. We are at a loss to explain these attempts of sectarics to obtain fellowship with those whom they abuse and denounce habitually as old fogies, bigots, teachers of error and workers of mischief, and even as man-slayers through their method of practice. Every sensible person knows that homeopathy was nurtured from its birth on antagonism to regular medicine, and that rank hostility to the regular profession has been its constant watchword. One would suppose that a class of people bitterly hostile to another class, holding them in contempt and claiming for themselves superior attainment and merit, would be glad to stand aloof and shun association with their deluded and bigoted inferiors. What if a strait moralist or a professor of religion should ask admission to, and affiliation with a club of gamblers, and cry persecution because his application was rejected? What if a Roman Catholic congregation should refuse to admit into its ranks a Unitarian or a Methodist unchanged in faith and unwashed of his heresies? All the world would say the Catholics were right and the applicant a fool or crazed. And so would people think if a Catholic should knock at the door of a Presbyterian Church and ask admission and fraternization, still adhering to his old faith and continuing to believe in the Pope and to worship the Virgin. Not a whit more consistent are homeopaths in asking admission to the professional fold without renouncing their peculiar and exclusive dogma, and the unscientific and preposterous notion of increasing the activity of remedies by reducing the dose to the millionth or even much beyond the millionth part of a millionth of a grain or drop.

We observe that a prominent newspaper in Liverpool takes the part of the poor, persecuted similibus men, and condemns the rejection of the only school of physicians founded on a purely scientific basis by an association whose members have no scientific methods, but whose practice is wholly empirical. The editor must have some peculiar notions as to what constitutes a science. It were well for him to learn something more in regard to the subject. Until he does so he had better practice on the old maxim, *ne sutor*, etc.

**Treatment of Measles.**—D. Maclean, M.D., Glasgow, physician to the Glasgow Public Dispensary Chest and Throat Clinic, in the Lancet, says: "The greatest action of the disease, as we all know, is upon the mucous membrane of the lungs, and it is from its action there we have the immediate cause of the ensuing death, or the prolonged ill-health afterward. We have, thus, clearly set before us the line of action to follow: (1) To relieve the congestion of the mucous membrane, which is the immediate cause of danger, and (2) to destroy or reduce the violence of the disease itself. This I have been in the habit of doing, I believe successfully, by giving (say to a child two or three years of age) a teaspoonful in water of the following mixture every three hours: Ipicacuanha wine, half a drachm; syrup of squills, half an ounce; quinine, two grains; acetate-of-ammonia solution to two ounces. Of course the quinine is increased according to age. We have, thus, in this mixture a stimulating expectorant and diaphoretic to relieve the tension in the mucous membranes and the skin, and also in the quinine a specific to destroy or abate the violence of the primary ferment. It may be necessary to add to or modify the form in which this plan of treatment is carried ont; as when the irritation and cough are persistently great; then the addition of a little tincture of hyoscyamus is all that is necessary. So with the quinine; sometimes the stomach is so irritable that it is necessary to omit it from the mixture; but as it is essential that it be introduced into the system for the destruction of the ferment, it can be administered separately by giving it in powder, mixed with saccharated carbonate of iron, which diminishes the irritant action of the quinine that takes place when the drug is given alone.

**Experimental Investigation into the Protective Power of Attenuated Tuberculous Material.**—These experiments were conducted by Dr. Falk, of Berlin, and were undertaken with the view of ascertaining whether animals in whom inoculation of tuberculous material had been practiced were thereby in any degree rendered insusceptible to a recurrence of the tuberculous process on repetition of the operation. Great difficulty was experienced in so modifying the material that it would not prove fatal on the first infection. The use of heat and chemical substances being impracticable, owing to the destructive character of these, the experimenter had recourse to a natural means of disinfection, i. e., to organic decomposition. This, as is well known, is antagonistic to infection; when, therefore, through continuous moistening of tuberculous tissues, a decomposition is set up in them, one may observe that the tubercle gradually loses its virulence. Using such material in which the decomposition had not advanced very far, Dr. F. introduced particles of it into the peritoneal cavity of animals; the result was not a general disease but only a local tuberculosis. This first inoculation surmounted and the local, cheesy abscess having undergone condensation and complete calcification, a second inoculation was attempted with fresh tubercles with infectious properties unimpaired. The results indicated that there is not the slightest ground for a belief in a protective inoculation in tuberculosis, for F observed that the animals which had already been inoculated were much more severely affected by the disease than those which had not undergone the process: and that the first inoculation, instead of being a means of protection, had decidedly increased the tendency to renewed disease by some sort of degenerative influence which it left after it.

The above experiments receive an explanation in the views advanced by Virchow and referred to in our last number. This author, it will be recollected, denies the infectiousness of tubercle, although conceding the ætiological significance of the bacillus tuberculosis. According to him this parasite acts only locally in parts in which it has secured a lodgment, and constitutional involvement is the result purely of processes which it sets up therein. Hence, the effect of inoculation is simply cumulative—merely adding to the contagium already present—just as planting a number of the itch insects over the surface of a person affected with scabies would do.

But it appears to us that, viewing the matter from the standpoint of the clinician, the difference is one rather of degree than kind, and it matters little to an individual whether a bacillus tuberculosis, inspired in the breath, causes a primary constitutional infection or not, provided it tends almost invariably, like acknowledged infectious processes, to lead to a fatal result.—*Maryl'd Med. Jour.*

**Radical Cure of Hernia.**—In two clinical lectures in the Indian Medical Gazette, Dr. R. McLeod describes the method of operating for the radical cure of hernia, which he has practiced in several cases during the last two years, and gives a table of the cases, with details of the results. His plan of operation—not peculiar to himself—is to isolate the sac quite up to the internal ring, there place a catgut ligature around it moderately tight, and tie the same thread at intervals of half an inch lower down the sac three times more. He cuts off the sac below the lowest ligature. With a Wood's needle he then stitches the inner pillar and conjoined tendon to the outer pillar and Poupart's ligament, leaving the ligatured neck of the sac as a plug in the inguinal canal. The wound treatment is antiseptic, and Dr. McLeod lays great stress on this point, insisting that the operation is not justifiable unless this precaution is taken. In all, Dr. McLeod has performed this operation twenty-eight times, with a total mortality of six. Of these cases, seven were patients with strangulation of the hernia, and this operation was done as a sequel to ordinary kelotomy; of these, one died of collapse eight hours after the operation, and another ten hours after the operation of laparotomy, performed five days and a half after the first operation on account of persistence of the symptoms of obstruction. In four patients the hernia complicated large scrotal tumors requiring operation. Two of these patients died from septicæmia, but in neither of them was there any peritonitis or mischief in the seat of the operation upon the hernia. The remaining seventeen were cases of reducible hernia, and among them there were two deaths—one from pyæmia, and one patient, who was removed from the hospital ten days after the operation, died two days later, probably from carbolic acid poisoning. These cases do not show the operation to be one attended with serious risk, if performed antiseptically. Four of the six fatal cases, in two the operation cannot be credited with causing the mortal shock. In the two cases fatal from septicæmia, the poison appears to have been absorbed from the scrotal, and not from the inguinal, wound. The other two deaths are directly traceable to the operation. Evidently it is not a procedure to be entered upon lightly, or without clearly explaining the risks to the patient. But whenever kelotomy has to be performed, it would appear to be wise, if possible, to supplement it by an attempt to procure a permanent cure of the affection, for this adds nothing to the danger of the original step, and, indeed, by closing the track into the peritoneal cavity, possibly lessens one of its perils.

**Absorbent Cotton as a Dressing.**—For over a year I have been using absorbent cotton as a dressing for the umbilical cord. I have been pleased beyond my expectations. I have had, during that time, not a single bad result—the cord coming off clean and smooth, not even the sign of a granulation. The skin around was not irritated in the least. My position as attendant upon the Christian Home here, a home for unfortunate girls, gives me a good opportunity to try it. Even with the inexperienced girls as nurses I have had no bad effects. When the child is ready to be dressed, I take a liberal piece of cotton and envelope the cord well. I use no grease of any kind. Dress the cord only once. Do not allow the dressing to be disturbed by the nurse looking at the cord each time the child is dressed. I thus prevent all pulling on the cord. The cord comes off in from three to four days, leaving a perfect navel. There is not, at any time, the slightest odor. I have used it in some twenty cases. I hope the members of the profession will give it a trial. It will, I think, in a number of cases, prevent umbilical hernia by preventing pulling, which occurs more or less in every case when cloth is used. It also gives gentle and equal pressure over the navel.—*W. D. Babcock, M.D., in Obstetric Gazette.*

**What is a Couvreuse?**—The London Lancet describes a pseudo invention called the couvreuse, which was suggested by the incubator for poultry. It is a plain wooden box about two and a half feet square and two feet four inches deep. A reservoir in the bottom of the box holds ten or twelve gallons of water, which is supplied from a boiler outside, and kept warm by a common lamp. Over the water tank is a bed of saw dust to retain the heat. A basket or cradle rests on the saw-dust bed, and will hold two infants. The cradle can be withdrawn by a side door. Over the box is a glass covering with a thermometer so placed as to indicate the temperature, which is kept about eighty-six degrees F. The infants can be seen at any time through the glass. The ventilation is said to be good, and though the children are taken out regularly to be fed and washed, they do not take cold. So successful has been the couvreuse that it is to be supplied to all the hospitals of France. Further, a small portable one is made, to be carried from house to house where it may be wanted.—*Pac. Med. Jour.*

**Injection of Morphia for Strangulated Hernia.**—In the Vratch Dr. Zolotnitzky reports a case of femoral hernia, strangulated for eighteen hours. All manipulation proving unsuccessful, he injected subcutaneously one-fifth of a grain of acetate of morphia in the neighborhood of the hernia. After one hour the tumor had nearly disappeared and a single touch of the finger completed reduction.—*Med. and Surg. Rep.*

**Salicylic Acid in Cerebro-Spinal Meningitis.**—Dr. Ramsay, in St. Louis Cour. Med., says:

1. The analogy existing between rheumatism and cerebro-spinal meningitis would suggest, and be good reason for the use of similar remedies in both diseases.

2. Salicylic acid being the best remedy, almost a specific in the treatment of acute articular rheumatism, would be a strong indication for its use in cerebro-spinal meningitis.

3. It produces a marked reduction in the temperature; the fever being thus lowered, the tissue-destruction and the onward progress of the inflammation is checked, thereby giving the patient rest.

4. It controls the intensely annoying metastatic pains of head, back, elbow and knee, giving the patient ease.

5, It exerts a direct influence for good over the inflammation itself, and can be taken in frequent large doses without bad effect; having given a boy fifteen years of age half-drachm doses every four hours for three or four days, with the only result of a great benefit in all the symptoms connected with the disease, is, I think, conclusive evidence of its harmlessness.

6. Its good effects are soon apparent, and it does not interfere with the use of other measures of relief, as ice, blisters, etc.

7. The best mode of using the remedy is to administer large doses frequently. For adults begin on doses of 15 grs. repeated every two hours, and increase the dose as may be found necessary to obtain the desired effect to ℈ ij, at intervals of two hours, if need be. When the disease is under control, which will be determined by the reduction in temperature, relief of pain and placid countenance, decrease the dose, give at longer intervals, but still continue the use of it in small doses as long as the least symptom is present indicative of the disease.

Having never heard nor read of salicylic acid being used in the treatment of cerebro-spinal meningitis, and my good success with its use in this fearful epidemic being afterward verified by Dr. J. B. Weever, of this place, I hope to induce others to give this remedy a trial, and by so doing I think they will be enabled to see very happy effects from its use, and thereby be highly gratified with the results.

**Glycerine after Bathing.**—Like the ancient Romans, D. S. Troy, of Montgomery, Ala., (Popular Science News,) has taken to using glycerine after bathing, with most happy results. He puts one drop of attar of roses in two ounces of glycerine, and rubs the body after bathing. The result was charming: it left the skin soft, with all of its functions in as full operation as before the bathing, and with a delicious sensation of perfect cleanliness. He has been using it for four or five years regularly, leading a life more sedentary than ever, and it seems to supply to the skin a vigor similar to that resulting from muscular exercise. It has been of great benefit to his general health and personal comfort. No care is required in rubbing it on, except to see that it is applied to all the skin except the face, and particularly to the soles of the feet. If any remains after the rubbing, it can be readily wiped off with a towel.—*Med. and Surg. Journal.*

**The Cholera Germ.**—News from Berlin, dated February 19, states that the German Sanitary Commission which was sent out to Egypt by the Imperial Board of Health to study the nature and causes, etc., of cholera, and which, after finishing its labors at Alexandria, was ordered to continue its investigations in India, has just forwarded a report from Calcutta, where it arrived some two or three months ago. The Commission discovered the cholera germ in a water-tank at Calcutta, and found in the suburban village where the cholera appeared the same microscopic organism which had been discovered in the lower intestines of cholera victims in Egypt.—*Med. and Surg. Rep.*

**International Medical Congress.**—The meetings of this Congress will begin at Copenhagen on August 10. A steamer will leave Hull (England) on August 2d and 9th for Copenhagen; and on August 5th a steamer will leave Leith (Scotland) for Copenhagen. Both these places (Hull and Leith) can be reached on any day by leaving Belfast on the previous evening by the cross-channel steamers. Visitors, after attending the meeting of the British Medical Association in Belfast, will have ample time to travel to Copenhagen for the Congress.—*Med. and Surg. Rep.*

**Good Advice.**—"Merit and patience then, my boy," says the Indiana Medical Journal, in the guise of a father's letter to a son just about to begin the practice of medicine, "is the course to pursue, in order to gain reputation and a large practice in the shortest possible period of time. These mushroom reputations have no solid foundation and are 'no good.' Let me entreat, then, in conclusion, to make haste slowly; to stick to your legitimate duties; to mind your own business; "whatever thy hand findeth to do, do it with all thy might." If you intend to make of yourself a physician, be a physician; keep apace with the times and the collaterals of your science as well as you can; cultivate good common sense and sound judgment; beware of the voice of flattery; be honest and truthful; scorn deceit. Do all these things, and you will have no occasion to advertise in the newspapers in order to secure a reputation or a large practice.

**It** is said that a small dose of morphine given with quinine is the best thing to counteract the unpleasant cerebral symptoms of the latter.

**Hippurate of Soda.**—In herbivorous animals the renal excretions rarely contain uric acid, but hippuric acid is always present. Uric acid is probably formed at one stage, but the presence of hippuric acid in considerable quantity effects its decomposition. Hippuric acid forms salts which are extremely soluble.

To approximate, therefore, the excretions from the kidneys of man to those of the herbivora, is to make an important step towards the prevention or removal, as the case may be, of the cause of diseases which arise from the defective elimination of uric acid. This may be attained by the employment of such a salt as hippurate of soda.

Dr. Garrod says: "There is no doubt that if hippurate of soda be added to a blood serum which shows the presence of a urate, the latter is soon removed from it."

(*1.*) *Powders.*—The hippurate of soda itself, dispensed in powder form, keeps quite well in paper. Combinations of the salt with lithia carbonate and citrate and bicarbonate of potash and soda, put up in powders in the usual way and kept for a fortnight, were found, on examination, to be in as good condition as when prepared.

(*2.*) *Mixtures.*—Like all alkaline salts, the taste of the hippurate of soda is disagreeably saline. I have tried a number of combinations with the object of rendering its administration as pleasant as possible, and the results mhy be briefly stated.

Chloroform water or spirit of chloroform seems to make it more disagreeable, rendering it almost nauseous.

Infusion of calumba disguises the saline taste, and, where the bitter is not an objection, affords an eligible vehicle.

The most agreeable mixtures, however, are obtained by employing syrup and peppermint water, or glycerin and cinnamon water.

The following examples may suffice:

(1.) ℞. Sodæ hippurat........................gr. 80,
Lithiæ carb........................gr. 24,
Glycerin........................ʒ iv,
Aq. cinnam........................ ad ℥ viij.

M. Sig. One-eigth part for a dose.

(2.) ℞. Sodæ hippur........................ ʒ ij,

Potass. citrat........................ ʒ iij,

Syrupi........................ ʒ vj,

Aq. menth. pip ........................ ad ℥ vj.

M. Sig. A tablespoonful for a dose.

The addition of an alkaline carbonate or citrate as given in the foregoing is desirable, so as to imitate the condition of the renal excretion of the herbivora, which is alkaline, that of man being usually acid.

The salt is very soluble, fifty grains dissolved in thirty minims of water forming a syrupy liquid. The dose may be from ten to fifteen grains.—*Phar. Jour. and Trans., Dec. 29, 1883.*

**Antral Abscess.**—Dr. Marshall (Chicago Medical Society Journal of American Medical Association) says:

Antral abscess is a much more serious difficulty than the affection just described, the former causing at its inception rigors and fever, and involves much more of the adjacent structures, periosteum, etc. In antral abscess the pus may be retained between this membrane and the bone, causing absorption of the walls of the antrum; and it may open into the mouth, orbit, or through the external surface, and cause hideous deformity. Extensive necrosis of the maxilla often follows, and it may extend to the ethmoid, lachrymal, palatine, and inferior turbinated bone, with cerebral abscess, unless early recognized and interfered with by surgical means.

When a dental abscess opens into the maxillary sinus, there may be danger of septic poisoning, if the secretions become foul and no other outlet is present. If this should occur it will be attended, of course, with serious constitutional symptoms. When it escapes into the nose the discharge will be unilateral, in its always being from the same nostril, and most abundant when a patient is lying upon the opposite side.

After the cases, with the symptoms, history, etc., etc., had been cited, the paper concluded with the treatment, which consists in syringing the cavity of a tooth and abscess through the pulp canal with warm carbolized water, 2 grs. to the ounce, once daily, and packing the tooth with carbolized cotton, and sealing it in with gutta-percha stopping to exclude external moisture. Sometimes the buccal roots can be explored with a fine probe, by passing it upwards for an inch and three-quarters. Water can be thrown into the antrum of Highmore through the roots of the teeth, and the fluid will escape into the nose. A tooth always needs to be extracted, if not already missing, and we then discover, most likely, that the pus will escape into the mouth through the alveoli, and that the ends of the buccal roots are somewhat rough and portions already have been removed by absorption. A few weeks' time of treatment in this manner usually results in a most satisfactory improvement or entire recovery of the patient."

**Dr. B. W. Richardson on the Morphia Habit and its Treatment.**—Dr. R.'s communication, read before the Medical Society of London, referred more particularly to the hypodermic injection. A considerable proportion of the cases under his notice had been physicians. The dose varies from under one grain to fifteen grains a day. Under three grains a day, the symptoms were not of sufficient gravity to interfere with the discharge of the ordinary duties of life; but larger doses soon began to cause marked symptoms. He had known as much as twelve grains a day injected over long periods, and had heard of a patient who used eighteen grains (the symptoms from 3½ gr. doses, as described by an habitue himself were here given); the more serious symptoms induced by deep indulgence in the habit were, ordinarily, intensified appetite for the drug, loss of power of the will, confirmed apathy without the ordinary toxic effects of opium, failure of all the mental faculties, together with general failure of voluntary and involuntary muscular power. The objective symptoms were those of diminished vitality generally; bending of the body and the signs of premature old age. Immediately after the dose, the temperature might be noticed to be commonly raised one or two degrees at least, until the habit had been long established; irregularity occurred as the effect of the dose was passing off. The danger incident to the practice was the next topic brought forward, qualified by the observation that, in certain forms of fetal diseases, the habit, with all its drawbacks, might sometimes be useful; might sometimes make existence easier, and prolong life. This, however, was the exceptional part of the practice, and required to be carefully separated from the general. The author believes that the symptoms produced by morphine and by opium-smoking are different, the narcotic effects being more pronounced in the latter. The total withdrawal of the drug effected in from seven to twenty-one days is the only natural and safe mode of cure. If the quantity be small and the habit be not of long duration, it might be advisable to suddenly withdraw, but when well established he adhered strongly to the above opinion.—*British Med. Jour.*

**Anæsthesia by a Mixture of Morphia, Atropia and Chloroform.**—Dr. C. Aubert communicates to the Societe de Biologie (Comptes Rendus) the results which he has obtained in his surgical practice by preceding the use of chloroform by the injection of

| | |
|---|---|
| Morphia chlor. hydrat. | 10 centigr., |
| Atropia sulph. | 5 milligr., |
| Aqua dist. | 10 grams. |

He uses 1½ grammes of this solution 20 or 30 minutes before beginning the inhalations. In children he uses not more than one gramme. He has used this method in more than 60 cases, and, as far as the atropia is concerned, has never seen the slightest disagreeable symptom, no dilatation of the pupil, perhaps a little dryness of the throat, and considers that these agents furnish great security against the danger of cardiac syncope following chloroform inhalations. The advantages claimed are: First, the security. Second, the greater rapidity with which anæsthesia is produced. Third, the absolute quiet of the patient. Fourth, the facility of awakening. Fifth, the abscnce of subsequent discomfort and vomiting.

As to security, he refers to one case where in an aged subject there existed adhesions of the pleuræ, extensive pulmonary emphysema, marked mitral insufficiency, interstitial nephritis and purulent cystitis, as proven by the autopsy three weeks later; this method was followed, and there was no perceptible influence upon the pulse. It is true that there was cyanosis which required the use of artificial respiration for two minutes. He accepts, therefore, the views of MM. Morat and Dastre, that atropia diminishes the excitability of the pneumogastric.

As to the rapidity of its action: out of 40 patients 4 came under its influence in 1 minute, 5 in 1½ minutes, 12 in 2 minutes, 6 in 2½ minutes, 2 in 4 minutes, 4 in 5 minutes, 1 in 6 minutes, 1 in 7 minutes, and 1 in 10 minutes.—*Jour. Ame. Med. Association.*

**Effects of Strong Light upon the Eye.**—Little ("Ophthalmic Review," July, 1883), reports two cases showing the effect of strong light upon the eye. The first was the case of a gentleman whose right eye had been exposed suddenly and for a moment to a very strong electric light. He was blinded for several minutes, and he suffered from intolerance of light and severe headache for some days. He complained of a mist and dark specks before the right eye, with vision of 20.50. There was a distinct haziness over the optic disc and neighboring retina. The second case was of blepharospasm caused by lightning. A gentleman, aged fifty-six, while walking, was struck down by lightning, but was not rendered insensible. On rising, he found he could not open his eyes. Two days later Little saw him, and found marked blepharospasm in both eyes. He said his eyes were hot and painful. He remained in this condition for thirteen days, when he opened his eyes partially. He then steadily improved and ultimately recovered, with good vision and a normal fundus.—*N. Y. Med. Record.*

**Succinate of Iron in Biliary Colic.**—Dr. Jas. A. Stewart, of Baltimore, revives the claim that the hydrated succinate of the peroxide of iron is efficient in the treatment of gall-stones. He reports one case in which a patient, a lady of forty, who had suffered for three months and was greatly emaciated, recovered health rapidly under drachm doses of the succinate. There had been no trouble for two years.—*Louisv. Med News.*

**Gelsemium for After-Pains.**—After-pains are sometimes exceedingly rebellious, hence our readers will be pleased to learn that Dr. Holt (New York Medical Journal, December 8, 1883), has used the fluid extract of gelsemium, in doses of a fraction of a drop, frequently repeated. In the case of a patient in whom opium was not tolerated, and where all other remedies had been tried, the relief from gelsemium was prompt and decided.—*Med. and Surg. Rep.*

**Iodoform in Ophthalmic Therapeutics.**—Vossius (Archiv fur Ophthalmologi, xxix, 1) recommends iodoform in all ulcerative processes of the cornea, especially in ulcus serpens. The earlier the case applies for treatment, the more rapid and favorable is the effect. Its use is not contra-indicated by any complications of the iris. He also advises its use as an antiseptic in all superficial and deep, accidental and artificial wounds of the conjunctiva and cornea, as well as in wounds of the sclera. He has but rarely noticed any evil effects from its use, and never any abscesses of the conjunctiva; nor has he ever met with the amblyopia spoken of by Hirschberg, and attributed by the latter to the use of this drug.—*N. Y. Med. Record.*

